# An ensemble model for predicting dispositions of emergency department patients

**DOI:** 10.1186/s12911-024-02503-5

**Published:** 2024-04-22

**Authors:** Kuang-Ming Kuo, Yih-Lon Lin, Chao Sheng Chang, Tin Ju Kuo

**Affiliations:** 1 Department of Business Management, National United University, No.1, 360301 Lienda, Miaoli Taiwan; 2 Department of Computer Science and Information Engineering, National Yunlin University of Science and Technology, No. 123, University Road, Section 3, 64002 Douliou, Yunlin Taiwan; 3 Department of Emergency Medicine, E-Da Hospital, Kaohsiung City, Taiwan; 4 Department of Occupational Therapy, I-Shou University, Kaohsiung City, Taiwan; 5 Department of Computer Science and Information Engineering, National Taitung University, 369, Sec. 2, University Rd, Taitung, Taiwan

**Keywords:** Base-learner, Emergency department disposition, Ensemble learning, Meta-learner, Predictive disposition method

## Abstract

**Objective:**

The healthcare challenge driven by an aging population and rising demand is one of the most pressing issues leading to emergency department (ED) overcrowding. An emerging solution lies in machine learning’s potential to predict ED dispositions, thus leading to promising substantial benefits. This study’s objective is to create a predictive model for ED patient dispositions by employing ensemble learning. It harnesses diverse data types, including structured and unstructured information gathered during ED visits to address the evolving needs of localized healthcare systems.

**Methods:**

In this cross-sectional study, 80,073 ED patient records were amassed from a major southern Taiwan hospital in 2018–2019. An ensemble model incorporated structured (demographics, vital signs) and pre-processed unstructured data (chief complaints, preliminary diagnoses) using bag-of-words (BOW) and term frequency-inverse document frequency (TF-IDF). Two random forest base-learners for structured and unstructured data were employed and then complemented by a multi-layer perceptron meta-learner.

**Results:**

The ensemble model demonstrates strong predictive performance for ED dispositions, achieving an area under the receiver operating characteristic curve of 0.94. The models based on unstructured data encoded with BOW and TF-IDF yield similar performance results. Among the structured features, the top five most crucial factors are age, pulse rate, systolic blood pressure, temperature, and acuity level. In contrast, the top five most important unstructured features are pneumonia, fracture, failure, suspect, and sepsis.

**Conclusions:**

Findings indicate that utilizing ensemble learning with a blend of structured and unstructured data proves to be a predictive method for determining ED dispositions.

**Supplementary Information:**

The online version contains supplementary material available at 10.1186/s12911-024-02503-5.

## Introduction

Healthcare systems face a myriad of challenges, such as an aging population and an increasing demand for quality health services. According to the United Nations (UN), for example, the global population of aging adults (aged 65 and older) is expected to grow significantly in the upcoming decades. The UN’s Department of Economic and Social Affairs [1] estimated that this age demographic will increase from 727 million in 2020 to 1.5 billion in 2050, representing a rise from 9.3 to 16% of the world’s total population. This demographic shift poses various challenges and opportunities for social and economic development [1], and the healthcare sector is no exception. Furthermore, the substantial repercussions of recent infectious diseases such as COVID-19 are exerting immense pressure on multiple facets of healthcare professionals’ responsibilities [2, 3], potentially even influencing the delivery of healthcare services. Seen from these perspectives, it becomes evident that the healthcare sector will persistently confront evolving, if not daunting, challenges in the years ahead.

One of these challenges involves the serious overcrowding witnessed within the Emergency Department (ED). This crowding dilemma in ED has transcended national boundaries to become a global concern for hospitals across the world. ED overcrowding in fact has made much impact on the safety and quality of patient care, according to prior reviews [4, 5]. The solutions for ED overcrowding that have been reported emphasize optimizing the balanced flow within the ED, such as the implementation of timed patient disposition targets [5] or predicting the ED workload [6]. An emerging trend in this context is the potential of machine learning to predict ED dispositions, which could offer significant benefits regarding throughput.

Currently, numerous studies have developed predictive models using machine learning techniques to predict patient dispositions in the ED. These predictive models utilize various types of data, including structured information like demographic details and vital signs [7–9], unstructured data like triage notes and chief complaints [10–12], or a combination of both structured and unstructured data [13–15]. While these studies have significantly contributed to our understanding of ED dispositions, most of the models they build predict only two dispositions at a time, such as discharge vs. admission, which may not always be practical whenever there are more than two possible ED dispositions. Furthermore, the potential of ensemble learning has not been fully explored in these studies, with the exception of [16–18]. Ensemble learning combines multiple individual classifiers / regressors to achieve better classification / regression performance than with each one separately [19].

The primary objective of this study is to construct a predictive model for the dispositions of patients in ED based on different types of data. To be more precise, we develop an ensemble learning-based model to forecast multiple outcomes of ED patients simultaneously, harnessing both structured and unstructured data gathered when patients seek treatment in the ED. Our study has the potential to make two contributions. Firstly, it provides a practical solution for the early prediction of multiple dispositions yet to take place in the ED. This enhances the ED’s ability to proactively manage available healthcare resources, allowing healthcare professionals to easily predict potential outcomes for ED patients without the need to consider multiple conditions or to use different predictive models. Secondly, our research employs ensemble-learning techniques to construct a predictive model that incorporates both structured and unstructured data. This approach sheds light on the effective application of ensemble learning across diverse data types to forecast patient dispositions in the ED.

## Related work

In the past, numerous studies have focused on establishing predictive models for ED dispositions. The types of feature used in these studies [10–18, 20–28] include structured data (such as age, gender, etc.) and unstructured data (such as nursing notes, chief complaints, etc.). Among these studies are many that solely utilize structured data to predict ED dispositions [7–9]. However, the number of studies that solely employ unstructured data or combine structured and unstructured data to predict ED dispositions is relatively smaller (*see* Supplementary file A).

For instance, Lucini et al. [12] exclusively employed unstructured medical records, transformed through natural language processing into features, to predict the probability of emergency patients’ hospitalization. The results showed that the support vector machine performed the best, achieving an F1-score of 77.7%. The strength of this study lies in its clear demonstration of machine learning performance using unstructured data. Moreover, Lucini et al. [12] tested their models using seven algorithms and compared their performance results. One noticeable limitation is that they solely predicted hospital admissions and non-hospital admissions, omitting other ED dispositions taking place. Tahayori et al. [10] also utilized triage notes to predict patient hospitalization, revealing that a deep neural network (DNN) achieved an accuracy of 0.83 and an area under the receiver operating characteristic curve (AUROC) of 0.88. This study excels in its utilization of the Bidirectional Encoder Representations from Transformers model to process triage notes. Similar to Lucini et al. [12], Tahayori et al. [10] also focused solely on predicting patient admission or homestay, without having to explore other ED dispositions.

Other examples, such as the study conducted by Zhang et al. [20], involved the combination of demographics and reasons for visiting the ED to predict the likelihood of patient hospitalization. This was achieved by utilizing both logistic regression and DNN to build predictive models. The results indicated that models combining structured and unstructured data outperformed models using structured or unstructured data alone. A notable aspect of this study is its incorporation of both structured and unstructured features in model development. Additionally, Zhang et al. [20] compared the performance of models using structured, unstructured, and combined data to clearly illustrate the efficacy of these different feature types. However, one limitation is that they solely predict admission or transfer (to other hospitals), neglecting an investigation into other possible ED dispositions. Duanmu et al. [28] used demographics, vital signs, laboratory data, and chest X-rays to predict ED patient mortality, and the study results demonstrated that the predictive ability of models combining structured and unstructured data had higher AUROC and accuracy when compared to those using structured or unstructured data alone. The merit of this study is evident in that Duanmu et al. [28] utilized both structured and unstructured data to establish their model. What is particularly noteworthy is their use of chest X-rays instead of free-text reports. However, it remains important to mention that they solely focused on predicting incidences of mortality or non-mortality, leaving other outcomes unexplored.

These studies that utilize unstructured data to predict ED disposition provide us with a deeper understanding of the predictive capability of unstructured data for ED disposition. From these existing studies, several directions for further investigation emerge that could potentially enhance machine-learning performance in predicting ED disposition. Firstly, there are relatively few studies which predict multiple ED dispositions simultaneously using a multiclass approach, with the majority employing binary class methods to build predictive models [11, 25, 27]. From a practical perspective, the leading principle should be the ability to predict different ED dispositions in an easy and comprehensive manner, without requiring distinct prediction models for each disposition. Secondly, while research [16–18] has begun to explore the use of ensemble learning techniques, additional studies are needed to further accumulate knowledge on their application in predicting ED dispositions, given the significance of this topic. Considering the favorable performance of ensemble learning [19], employing ensemble learning for building predictive models of ED disposition could uncover its true potential performance.

## Methods and material

### Study population and setting

This study is a retrospective cohort study with the primary objective of predicting the dispositions of ED patients using both structured and unstructured data. The structured data primarily encompass patient demographics, vital signs, and physician-diagnosed conditions encoded as ICD-10-CM. The unstructured data includes the subjective section of SOAP (subjective, objective, assessment, and plan) notes and the preliminary diagnosis from the first physician encounter. The subjective section mainly comprises chief complaints, present illness diagnosis, and the patients’ past medical history.

The data for this study were obtained from a large teaching hospital located in southern Taiwan. The hospital has approximately 1,200 beds, with an average monthly ED visit volume of around 4,000 patients. The data collection period spans from 2018 to 2019. The patient data for the two years amounted to 57,751 and 56,744 cases, respectively. Data for patients under the age of 20 were excluded. Additionally, samples with vital sign measurements that fell beyond reasonable ranges were removed (e.g., respiration rate: 0–60). Furthermore, since the study objective is to predict ED dispositions using both structured and unstructured data, samples with missing data were also removed. After these exclusions, there were 40,667 and 39,406 patient cases remaining for the respective years, resulting in a total of 80,073 patient records on hand.

### Feature and outcome variables

The features used in this study were recommended by an ED physician (> 10 years of clinical experience, possessing a Master’s degree) and determination made based on relevant literature [14, 15, 20, 22, 25] (*see* Table 1). The features were categorized into three types: continuous, categorical, and text variables. Continuous variables include: Age, temperature, pulse rate, respiration rate, diastolic blood pressure, systolic blood pressure, and saturation of peripheral oxygen. Taiwan triage and acuity scale (TTAS), as defined by the Ministry of Health and Welfare of Taiwan, relies on vital signs, is guided by chief complaints, and considers physiological conditions. This system employs primary and secondary regulating variables to determine a patient’s triage level (with five distinct levels) and establishes relative safe waiting / observation times for patients at each level. These regulating variables encompass aspects like respiratory distress, hemo-dynamics, level of consciousness, body temperature, and degree of pain. TTAS is further divided into two primary systems: non-trauma and trauma. The non-trauma system comprises 14 categories, encompassing a total of 132 chief complaints, while the trauma system is subdivided into 15 categories, covering a total of 47 chief complaints. Triage codes are used to correlate with the chief complaints of patients and indicate the severity as assessed by attending nurses. Additionally, text variables encompass the subjective section of SOAP notes and preliminary diagnoses provided by physicians.

**Table 1:** Features included in this study

Feature format	Feature	Measurement	Description (Range)
Structured	Age	Numeric	≥ 20 years old
	Gender	Categorical	Male or female
	Temperature	Numeric	In Celsius (0–43.2)
	Pulse rate	Numeric	Beats per minute (0–220)
	Respiration rate	Numeric	Breaths per minute (0–60)
	Systolic blood pressure	Numeric	Millimeter of mercury (0–250)
	Diastolic blood pressure	Numeric	Millimeter of mercury (0–130)
	Saturation of peripheral oxygen	Numeric	Percent (0–100)
	Glasgow coma scale	Categorical	(3–15)
	ICD-10-CM	Categorical	26 classes coded from A– Z
	Taiwan triage and acuity scale	Categorical	Level 1: Resuscitation Level 2: Emergent Level 3: Urgent Level 4: Less urgent Level 5: Not urgent
	Triage code	Categorical	- Non-trauma: 14 categories with 132 chief complaints - Trauma: 15 categories with 47 chief complaints
Unstructured	Subjective and preliminary diagnosis	Text	Free text

*Note* ICD-10-CM means International Classification of Diseases, Tenth Revision, Clinical Modification

The outcome variables in this study comprise three categories: admission, discharge, and expiration. Admission denotes patients who were admitted to the hospital for further treatment or observation after their initial ED visit. Discharge refers to patients who were released from the ED after receiving some form of treatment. Expiration signifies patients who passed away before adjacent to leaving the ED.

### Experimental setup

**Fig. 1:**
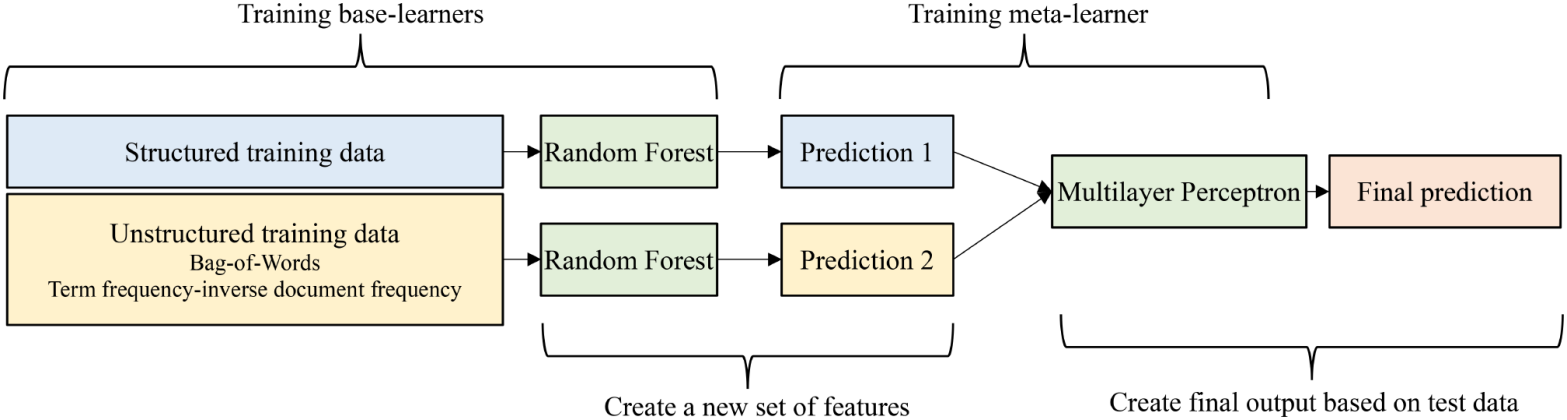
Diagram of the ensemble model flow

This study builds a patients’ ED disposition prediction model using ensemble learning. As Fig. 1 shows, the collected data, including structured and unstructured, was initially divided into training and testing sets in a respective 70 − 30 ratio. This study utilizes Random Forest (RF) as the base-learners and employs Multilayer Perceptron (MLP) as the meta-learner, leveraging their well-established performance. In particular, neural network algorithms have found widespread application across various disciplines, demonstrating strong performance [29–31].

We conducted a performance comparison among five algorithms—Random Forest, Adaboost, Logistic Regression, Support Vector Machine, and Naïve Bayes—prior to building the ensemble model. Among these algorithms, Random Forest demonstrated superior performance, particularly in handling structured data. Consequently, we chose Random Forest as the baseline model for our further analysis. The base-learners comprise two models built using RF, one using structured data and the other using unstructured data.

Structured data undergoes one-hot encoding for categorical variables, but numeric variables are not scaled for performance consideration. Unstructured data, on the other hand, is processed through both the bag-of-words (BOW) and term frequency-inverse document frequency (TF-IDF) techniques. (*see* Fig. 1). BOW converts words into numerical representations without considering semantic information, while TF-IDF also transforms words into numerical vectors by incorporating weighted information [32]. In Taiwan, ED physicians primarily write their clinical notes in English; therefore, translations will not be a concern. Text pre-processing is conducted, involving the conversion of uppercase letters to lowercase, removal of punctuation and stop-words, before performing BOW and TF-IDF transformations based on unigrams. Furthermore, abbreviations, misspelled words, or phrases with preceding negations are retained in this study because they may still contain relevant information after vectorization. The outcome variable, which consists of three categories, undergoes one-hot encoding.

To predict ED dispositions, the first RF model incorporates 237 features, while the second RF model incorporates 250 features. The output of the first and second RF models is located in either of the following formats: [100], [010], or [001], respectively. The predicted outputs from these two models are then combined to form new features (e.g., in the format of [100,100]), which are subsequently utilized as additional features with which to further train the MLP. The final model constructed by the MLP is validated using the testing data, generated in the same way as the new features created by the first and second RF models.

To ensure optimal performance of the predictive model, this study employs the random search method to find the best hyper-parameters for the base-learners and meta-learners for both the structured and unstructured data. For RF models, we tune two hyper-parameters including n_estimators and max_features. For MLP, we tune three hyper-parameters including the number of neurons, activation function, and optimizer. Table 2 shows the optimal hyper-parameters for both RF and MLP models.

**Table 2:** Model parameter setting

Algorithm	Setting
Random Forest (for structured data)	λ n_estimators: 1000 λ max_features: 237
Random Forest (for unstructured data)	λ n_estimators: 1000 λ max_features: 250
Multilayer Perceptron	λ One hidden layer with 64 neurons λ Activation function: relu λ Optimizer: Adam λ Loss function: categorical_crossentropy

### Performance measures

In machine-learning classification problems, the evaluation of the discrimination of the optimal solution is typically obtained from a confusion matrix (*see* Table 3). The values in the columns of the confusion matrix represent the predicted outcomes, while the values in the rows represent the actual outcomes. True Positive (TP) and True Negative (TN) respectively indicate the number of positive and negative instances correctly predicted. False Positive (FP) and False Negative (FN) represent the numbers of positive and negative instances incorrectly predicted [33]. From the confusion matrix, various metrics such as accuracy, area under the receiver operating characteristic curve (AUROC), precision, recall, and F1 score may be calculated using the formulas located in Supplementary file B.

**Table 3:** Confusion matrix

		Truth	
		Positive	Negative
Predicted	Positive	True positive (TP)	False negative (FN)
	Negative	False positive (FP)	True negative (TN)

Accuracy indicates the ratio of correctly predicted instances to the total number of instances. It is straightforward to compute and understand, and it is applicable to both binary and multi-class classification problems [33]. AUROC is a more robust measure of model performance with instances of unbalanced datasets [34], which aligns well with the scenario found in our study. AUROC measures a model’s ability to distinguish between classes by comparing the true positive rate with the false positive rate for each class combination or against all other classes across various threshold levels [34]. Precision represents the proportion of truly positive instances among those predicted as positive, while recall signifies the proportion of truly positive instances that were correctly predicted as positive. F1 score is then derived as the harmonic mean of precision and recall, aiming to provide a more representative metric. Given that our study involves a multi-class classification problem with unbalanced predicted classes, calculating AUROC, precision, recall, and F1 score using the micro method (aggregate the contributions of all classes to compute the average metric) is more suitable [35].

## Results

### Data characteristics

Regarding continuous features (*see* Table 4), out of the 80,073 patients examined, the median age is 57. The median temperature is 36.60, the median pulse rate is 87, respiration rate is 18, median systolic blood pressure is 134, median diastolic blood pressure is 80, and the median saturation of peripheral oxygen is 97.

**Table 4:** Characteristics of numeric structured features

Feature	2018 (*N* = 40,667)	2019 (*N* = 39,406)	Total (*N* = 80,073)
	Median (IQR)	Median (IQR)	Median (IQR)
Age	56 (39–70)	57 (39–71)	57 (39–70)
Temperature	36.60 (36.2–37.1)	36.60 (36.2–37)	36.60 (36.2–37)
Pulse rate	87 (75–100)	87 (75–100)	87 (75–100)
Respiration rate	18 (18–20)	18 (18–20)	18 (18–20)
Systolic blood pressure	134 (118–153)	134 (118–153)	134 (118–153)
Diastolic blood pressure	80 (70–90)	79 (69–90)	80 (69–90)
Saturation of peripheral oxygen	97 (96–99)	97 (96–98)	97 (96–98)

*Note* IQR means interquartile range

Regarding categorical features (*see* Table 5), the proportion of males is higher than females (53.25% vs. 46.75%). The Glasgow coma scale scores 15 points for the majority of cases (92.11%). The ICD-10-CM classification “Symptoms, signs and abnormal clinical and laboratory findings, not elsewhere classified” has the highest proportion (24.66%). The Taiwan triage acuity scale is predominantly at level 3 (73.82%). Triage code A03 (Disease of gastrointestinal system) has the highest occurrence (18.37%). The proportions of ED patients who are admitted, deceased, and discharged are 40.38%, 0.20%, and 59.42%, respectively. The five-number summary of the unstructured data consists of the following values: A median of 41, a first quartile of 15, a third quartile of 78, a minimum value of 1, and a maximum value of 1579.

**Table 5:** Characteristics of categorical structured features

	2018 (*N* = 40,667)			2019 (*N* = 39,406)			Total (*N* = 80,073)		
Attribute	Value	Frequency	%	Value	Frequency	%	Value	Frequency	%
Gender	Female	18,765	46.14	Female	18,668	47.37	Female	37,433	46.75
	Male	21,902	53.86	Male	20,738	52.63	Male	42,640	53.25
Glasgow coma scale	3	247	0.61	3	234	0.59	3	481	0.60
	4	101	0.25	4	77	0.20	4	178	0.22
	5	124	0.30	5	127	0.32	5	251	0.31
	6	170	0.42	6	133	0.34	6	303	0.38
	7	194	0.48	7	166	0.42	7	360	0.45
	8	229	0.56	8	208	0.53	8	437	0.55
	9	280	0.69	9	273	0.69	9	553	0.69
	10	541	1.33	10	462	1.17	10	1,003	1.25
	11	278	0.68	11	235	0.60	11	513	0.64
	12	247	0.61	12	212	0.54	12	459	0.57
	13	279	0.69	13	290	0.74	13	569	0.71
	14	604	1.49	14	606	1.54	14	1,210	1.51
	15	37,373	91.90	15	36,383	92.33	15	73,756	92.11
Top 10 ICD-10-CM	R	10,427	25.64	R	9,318	23.65	R	19,745	24.66
	S	7,657	18.83	S	7,544	19.14	S	15,201	18.98
	K	3,812	9.37	K	3,860	9.80	K	7,672	9.58
	J	3,600	8.85	J	3,729	9.46	J	7,329	9.15
	N	2,551	6.27	N	2,786	7.07	N	5,337	6.67
	I	2,315	5.69	I	2,228	5.65	I	4,543	5.67
	M	2,056	5.06	M	1,924	4.88	M	3,980	4.97
	T	1,811	4.45	T	1,652	4.19	T	3,463	4.32
	L	1,466	3.60	L	1,534	3.89	L	3,000	3.75
	C	744	1.83	A	712	1.81	A	1,409	1.76
Taiwan triage acuity scale	Level 1	2,671	6.57	Level 1	2,443	6.20	Level 1	5,114	6.39
	Level 2	4,672	11.49	Level 2	4,176	10.60	Level 2	8,848	11.05
	Level 3	29,585	72.75	Level 3	29,522	74.92	Level 3	59,107	73.82
	Level 4	3,700	9.10	Level 4	3,241	8.22	Level 4	6,941	8.67
	Level 5	39	0.10	Level 5	24	0.06	Level 5	63	0.08
Top 10 triage code (Note 3)	A03	7,627	18.75	A03	7,080	17.97	A03	14,707	18.37
	A04	5,347	13.15	A13	5,330	13.53	A04	10,590	13.23
	A13	5,091	12.52	A04	5,243	13.31	A13	10,421	13.01
	T12	4,157	10.22	T12	4,070	10.33	T12	8,227	10.27
	A01	3,247	7.98	A02	2,998	7.61	A02	6,216	7.76
	A02	3,218	7.91	A01	2,952	7.49	A01	6,199	7.74
	A06	2,155	5.30	A06	2,151	5.46	A06	4,306	5.38
	A05	1,585	3.90	A09	1,580	4.01	A09	3,098	3.87
	A07	1,575	3.87	A07	1,513	3.84	A07	3,088	3.86
	A09	1,518	3.73	A05	1,472	3.74	A05	3,057	3.82
Disposition	Admission	16,687	41.03	Admission	15,646	39.70	Admission	32,333	40.38
	Expire	83	0.20	Expire	81	0.21	Expire	164	0.20
	Discharge	23,897	58.76	Discharge	23,679	60.09	Discharge	47,576	59.42

*Notes* 1.ICD-10-CM initials: A: Certain infectious and parasitic diseases, C: Diseases of the blood and blood-forming organs and certain disorders involving the immune mechanism, I: Diseases of the circulatory system, J: Diseases of the respiratory system, K: Diseases of the digestive system, L: Diseases of the skin and subcutaneous tissue, M: Diseases of the musculoskeletal system and connective tissue, N: Diseases of the genitourinary system, R: Symptoms, signs, and abnormal clinical and laboratory findings, not elsewhere classified, S: Injury, poisoning, and certain other consequences of external cause, T: External causes of morbidity

2. Triage code: A01: Respiratory system, A02: Cardiovascular system, A03: Gastrointestinal system, A04: Nervous system, A05: Skeletal system, A06: Urinary system, A07: Ear, nose, and throat system, A09: Integumentary system, A13: General and other, T12: Limb injuries

3. Due to differences in the ranking of triage codes between 2018 and 2019, the ‘Total’ column represents the aggregated ranking based on the combined frequencies of triage codes from both years, presented in descending order

### Model building

In terms of model performance, when predicting ED dispositions using either structured or unstructured data alone, the unstructured data model (processed using BOW and TF-IDF) exhibited slightly better performance during the training phase, when compared to the structured data model. Among the unstructured data models, the TF-IDF model out-performed the BOW model. During the testing phase, the unstructured data model based on TF-IDF still out-performed the structured data model, while the structured data model’s performance was superior to that of the unstructured data model processed with BOW (*see* Table 6).

**Table 6:** Performance comparison of predictive models

	Training dataset						Test dataset				
		Accuracy (SD)	AUROC (SD)	F1 (SD)	Precision (SD)	Recall (SD)	Accuracy (95% C.I.)	AUROC (95% C.I.)	F1 (95% C.I.)	Precision (95% C.I.)	Recall (95% C.I.)
Random Forest (Structured data)		0.794 (0.005)	0.846 (0.004)	0.794 (0.005)	0.795 (0.005)	0.794 (0.005)	0.791* (0.784–0.798)	0.844* (0.838–0.849)	0.791* (0.784–0.799)	0.792* (0.784–0.799)	0.791* (0.784–0.798)
Random Forest (Unstructured data)	BOW	0.792 (0.008)	0.844 (0.006)	0.792 (0.008)	0.793 (0.008)	0.792 (0.008)	0.793* (0.786-0.800)	0.845* (0.839–0.850)	0.793* (0.786-0.800)	0.794* (0.786–0.801)	0.793* (0.786-0.800)
	TF-IDF	0.794 (0.005)	0.846 (0.004)	0.795 (0.005)	0.795 (0.005)	0.794 (0.005)	0.792* (0.785–0.799)	0.844* (0.839–0.849)	0.793* (0.786–0.799)	0.793* (0.787-0.800)	0.792* (0.785–0.799)
Multilayer Perceptron (Structured + Unstructured data)	BOW	0.939 (0.007)	0.973 (0.002)	0.896 (0.094)	0.942 (0.000)	0.936 (0.015)	0.937* (0.932–0.943)	0.971* (0.969–0.974)	0.906* (0.857–0.945)	0.937* (0.932–0.943)	0.937* (0.932–0.943)
	TF-IDF	0.936 (0.008)	0.972 (0.002)	0.849 (0.090)	0.938 (0.000)	0.933 (0.016)	0.938* (0.933–0.944)	0.972* (0.970–0.975)	0.896* (0.840–0.936)	0.938* (0.933–0.944)	0.938* (0.933–0.944)

*Notes* 1.BOW means Bag-of-Words, TF-IDF means term frequency–inverse document frequency, AUROC means area under the receiver operating characteristic, F1 means F1 score, SD means standard deviation, and C.I. indicates confidence interval

2. * indicates *p* < 0.001

Regarding the ensemble model combining structured and unstructured data, its performance in both the training and testing phases surpassed that of using either structured or unstructured data alone (*see* Table 6). For instance, overall AUROC increased from 0.8 in the training phase to 0.9, with similar trends observed in other metrics. As for the ensemble models using BOW and TF-IDF, their performances exhibited strengths and weaknesses in various evaluation metrics. The testing phase performances and training phase performances of individual structured data, unstructured data, and ensemble models showed minimal differences, indicating that overfitting is not an issue for the established models. Furthermore, we assessed the stability and reliability of test results by using 1,000 bootstrap resampling with the percentile method to obtain 95% confidence intervals [36]. *P*-values were then calculated based on these intervals [37], as shown in Table 6. Table 7 illustrates the evaluation metrics for each class for test datasets.

**Table 7:** Metrics for each class for test datasets

Model	Disposition	Accuracy	AUROC	F1	Precision	Recall
BOW	Admission	0.908	0.938	0.920	0.932	0.909
	Expire	0.738	0.940	1.000	1.000	1.000
	Discharge	0.958	0.938	0.970	0.940	1.000
TF-IDF	Admission	0.910	0.942	0.920	0.931	0.910
	Expire	0.691	0.957	1.000	1.000	1.000
	Discharge	0.957	0.942	0.970	0.942	1.000

*Notes* BOW means Bag-of-Words, TF-IDF means term frequency–inverse document frequency, AUROC means area under the receiver operating characteristic, F1 means F1 score

**Fig. 2:**
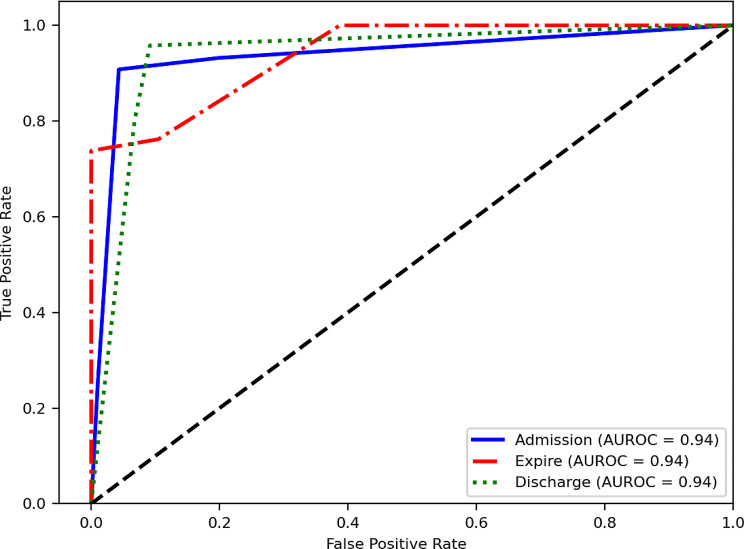
Area under receiver operating characteristic curve based on bag-of-words

**Fig. 3:**
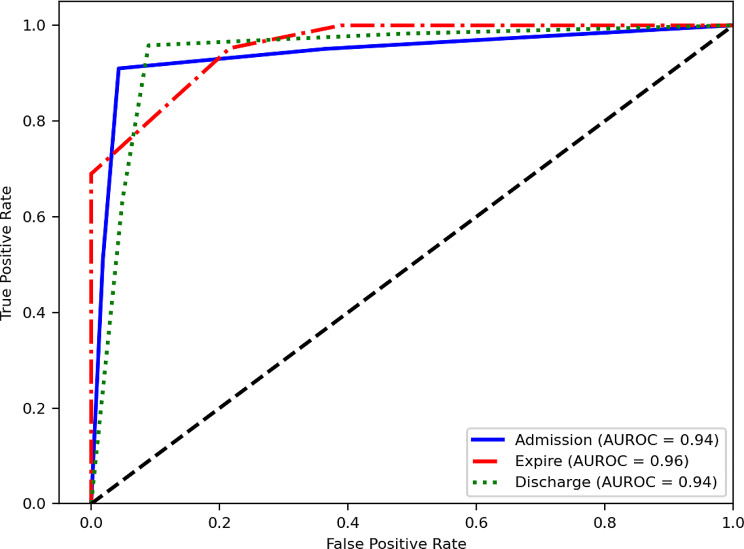
Area under receiver operating characteristic curve based on term frequency-inverse document frequency

**Fig. 4:**
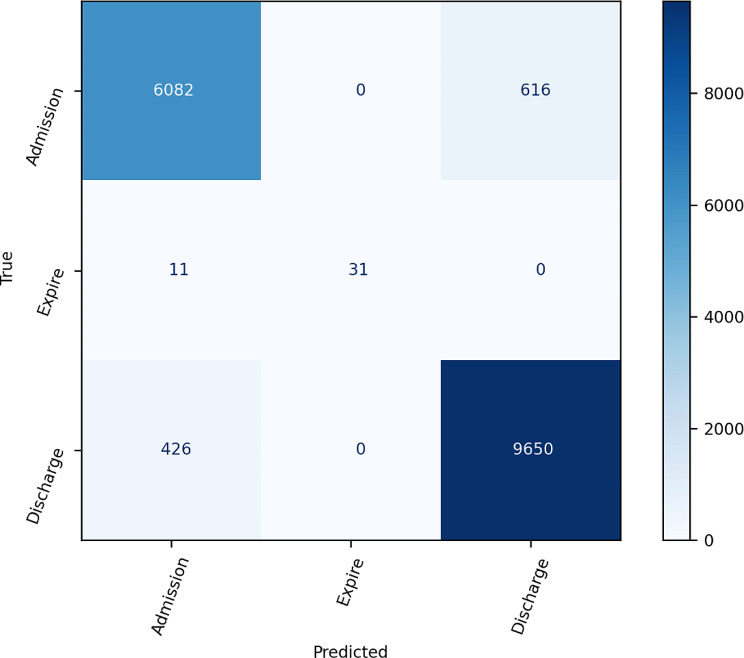
Confusion matrix based on bag-of-words

**Fig. 5:**
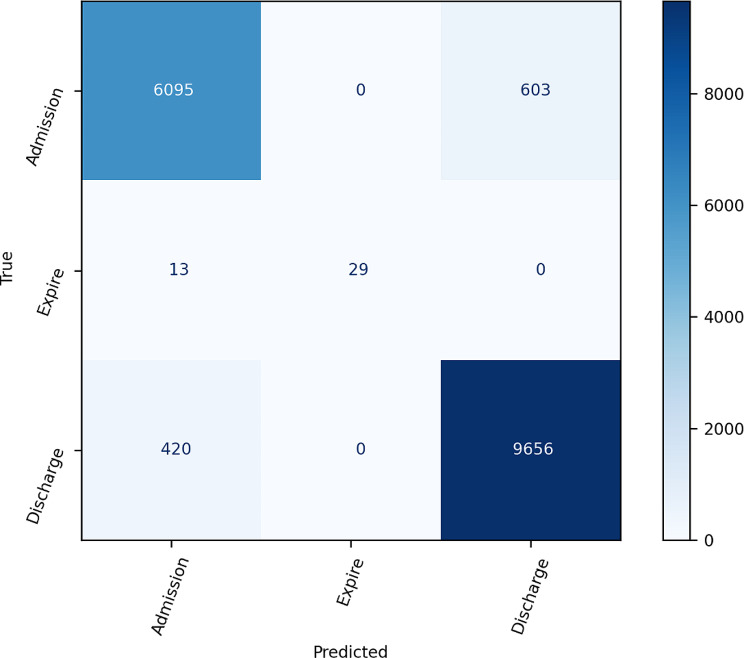
Confusion matrix based on term frequency-inverse document frequency

When examining class-specific AUROC values for the comparison of three ED dispositions, models constructed using the BOW method consistently demonstrated AUROC values of 0.94. This suggests comparable predictive capabilities across all three ED dispositions (*see* Fig. 2). Models established using the TF-IDF method showed slightly higher predictive ability for the expire disposition when compared to the other two dispositions (*see* Fig. 3). The confusion matrices generated by the ensemble models using the BOW and TF-IDF methods are shown in Figs. 4 and 5, respectively.

### Variable importance and model interpretation

**Fig. 6:**
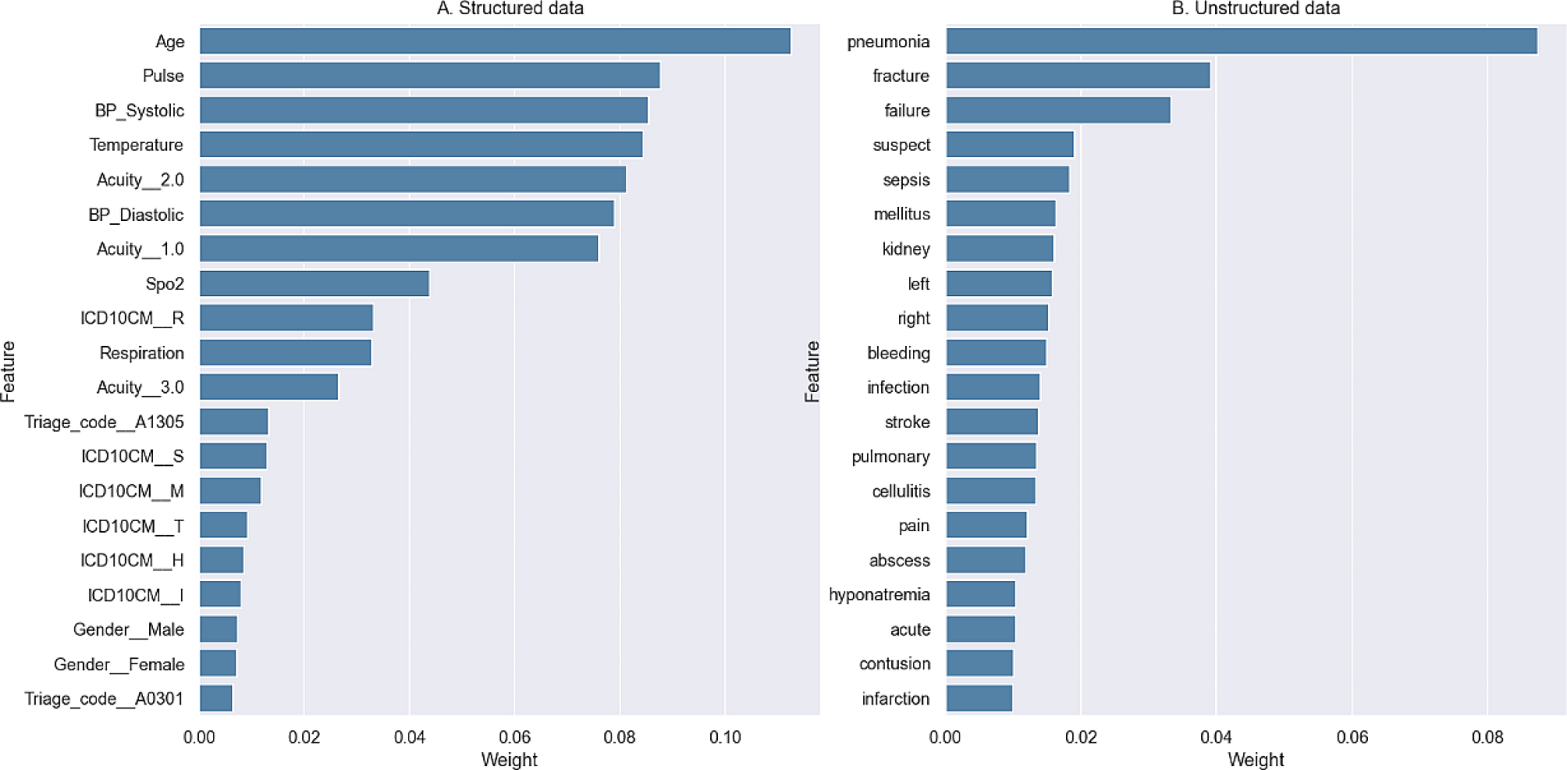
Feature importance of structured data (**A**) and unstructured data (**B**)

To understand the predictive nature of the model, this study employs Local Interpretable Model-agnostic Explanations (LIME) [38] to calculate the weights of structured and unstructured data features (*see* Fig. 6) and to explain the functioning of the predictive model itself. In terms of feature importance, the most crucial features in the structured data were age, followed by pulse rate, systolic blood pressure, temperature, acuity level, diastolic blood pressure, saturation of peripheral oxygen, ICD-10-CM, and respiration rate. In the unstructured data, the most significant features were pneumonia, followed by fracture, failure, suspect, sepsis, mellitus, kidney (left, right), and bleeding.

**Fig. 7:**
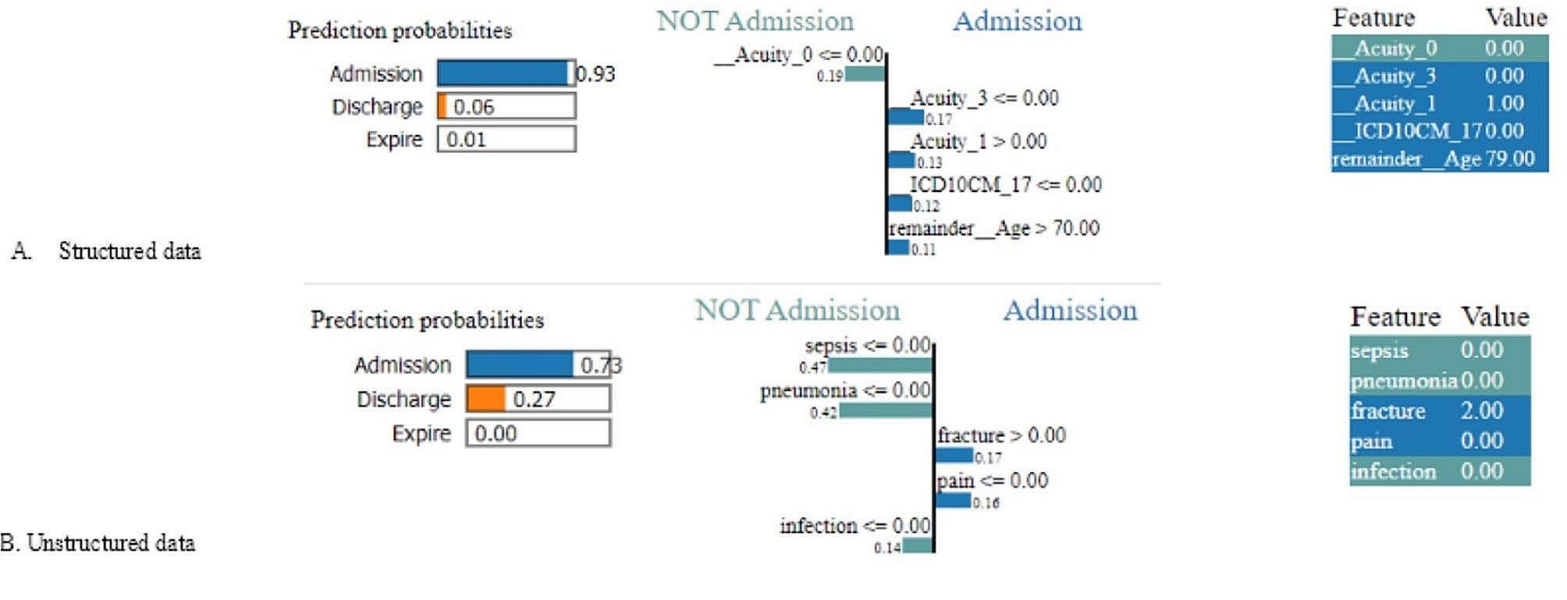
Explanation of prediction based on Local Interpretable Model-Agnostic Explanations

To illustrate how features influence model predictions, this study provides explanations for both structured and unstructured data. In this example, we use BOW to convert the unstructured data into a vectorized format. Figure 7 (comprising Fig. 7A and B) depicts predictions for individual samples.

In Fig. 7A, the left-most bar corresponds to the predicted probability, with the final prediction being “Admission” due to its probability of 0.93 in this example. The middle section of Fig. 7A illustrates the influence of features on the prediction outcome. Notably, conditions and features that contribute to an increased probability of predicting “Admission” include Acuity_3 ≤ 0.00, Acuity_1 > 1.00, ICD10CM_17 ≤ 0.00, and Remainder Age > 70.00. Conversely, features and conditions that diminish the prediction probability of “Admission” include Acuity_0 ≤ 0.00.

The right-most part of Fig. 7A displays the feature values for this example, offering insights into their impact on the predictive outcome. In this instance, the values (0 or 1) for features such as “Acuity_0,” “Acuity_3,” “Acuity_1,” and “ICD10CM_17” result from one-hot encoding, as these features are categorical. The feature “Age,” with a value of 79, is continuous; however, we set the LIME parameter discretize_continuous = True. This choice was made to facilitate more intuitive explanations by discretizing continuous features.

The same approach is applicable to interpret unstructured data, as shown in Fig. 7B. The prediction result in this case is “Admission” with a probability of 0.73, as indicated on the left side of Fig. 7B. Features such as “fracture” and “pain” contribute to an increased probability, while features like “sepsis,” “pneumonia,” and “infection” decrease probability, as illustrated in the middle of Fig. 7B. Since we use BOW to vectorize text data, the values of the text features represent their frequency of occurrence, as depicted on the right side of Fig. 7B. In this example, the text features “fracture” with a value of 2 and “pain” with a value of 0 contribute to the higher probability of the outcome “Admission.”

## Discussion

Based on the structured and unstructured data from ED visits in the years 2018–2019, this study constructed an emergency department discharge trend prediction model using ensemble learning. The results demonstrated that the predictive model’s performance, when combined with both structured and unstructured data, indeed outperformed the performance obtained when using structured or unstructured data singularly. The performance of unstructured data, whether processed using the BOW or TF-IDF method, was comparable. This study also identified significant and purposeful structured and unstructured features. Age and pneumonia emerged as two important features that may sincerely influence the discharge trend of ED patients.

This study combined both structured and unstructured data to predict the dispositions of ED patients. The overall model’s AUROC was approximately 0.97, and the individual AUROCs for predicting admission, discharge, or expiration were also 0.94 or higher. These results surpass the findings of previous studies that predicted ED patient disposition using structured and unstructured data [14, 15, 17, 21], some of which [15, 17] incorporated laboratory data not included as part of this study.

Furthermore, in comparison to other studies that used unstructured data, such as medical imaging, combined with structured data [25, 27, 28], the predictive performance of the machine-learning model constructed in this study was either superior or comparable in nature.

Ensemble learning is regarded as a promising machine-learning technique. Existing literature on building ED disposition models using ensemble learning based on unstructured data is still limited [16–18]. In this study, RF is employed to separately establish base-learners for both structured and unstructured data, with a MLP serving as the meta-learner. The overall predictive capability of the model was either higher or on par with previous studies that utilized ensemble learning [16–18].

Further, the outcomes considered in this study encompass admission, discharge, and expiration, constituting a multi-class classification problem. In prior research that focused on unstructured data, the emphasis was primarily on binary-class classification problems [13, 14, 27, 28]. In clinical practice, if the goal is to predict various ED disposition outcomes, it might necessitate the use of distinct predictive models. However, through the multi-class predictive model developed in this study, clinical practitioners can conveniently forecast potential dispositions for ED patients.

Regarding feature importance, estimated through the LIME, the important structured features in our predictive model include: Age, pulse rate, systolic blood pressure, temperature, acuity level, and diastolic blood pressure. The crucial unstructured features include: pneumonia, fracture, failure, suspect, sepsis, mellitus, kidney, left, right, and bleeding. In the context of structured features, previous studies [14, 15, 22, 25] also found that age, pulse rate, temperature, systolic blood pressure, diastolic blood pressure, and emergency severity level are all important predictors of ED Disposition.

### Theoretical implications

This study employs the ensemble learning method to establish an ED disposition predictive model, and the predictive performance obtained is satisfactory, indicating the genuine potential of ensemble learning in this context. However, there are still gaps in research involving ensemble learning applied to ED disposition prediction, particularly whenever incorporating unstructured data. Future studies could consider exploring various ensemble-learning strategies to develop ED disposition predictive models.

Most existing ED disposition predictive models are designed for binary classification problems, and there is a rather noticeable absence of models for multi-class classification. Given the number of possible ED dispositions, obtaining accurate predictive outcomes should be categorized as a multi-class classification problem. Future studies should explore the development of multi-class predictive models, which are likely to be more suitable for convenient clinical use in the ED. Even so, the expiration class has a significantly smaller number of samples when compared to the other two classes, and as such, the ensemble learning approach adopted in this study has the potential to effectively handle class imbalance, as highlighted by [39]. Future research might explore the utilization of random over/under sampling techniques as a means to address the challenge of class imbalance similar to the one existing in this study.

### Practical implications

The predictive model developed in this study has the capability to predict three dispositions concurrently: Admission, discharge, and expiration. This simplifies its use for ED clinical staff, eliminating the necessity for employing multiple distinct predictive models to forecast various dispositions. In addition, the important features identified in this research can function as valuable reference points for ED clinical staff when providing patient care. When combined with LIME’s model prediction explanation capability, it enables ED clinical staff to closely monitor changes in these salient features, which could potentially impact the severity of a patient’s condition. More specifically, healthcare professionals can utilize our model to predict the potential dispositions of patients arriving at the ED with more severe conditions and/or lower placement on the Glasgow Coma Scale. It is also significant to note that our model incorporates the LIME package, which effectively identifies key features contributing to the prediction, even for patients having shorter ED stays.

### Limitations and future directions

Our study has several limitations. The first, the samples collected were from only one hospital, which may limit the generalizability of the predictive model. Future studies may choose to collect data from more hospitals to reliably improve upon results. Second, no laboratory and image data were considered as part of this study, meaning that future studies may consider these different data and compare their performance with the structured and unstructured data used. Third, the model built in this study aims to predict the disposition of ED patients by the end of their ED visits regardless of what the duration of their visit may be. We did not limit the window of features used for the prediction task to a specific time-frame, such as with the first hour of the ED visit. Future research may identify such a specific time-frame to focus results according to severity or the nature of the visit. Currently, we do not process phrases with preceding negations. However, for future research, it may be worthwhile to consider incorporating rules or methods that can identify negations that may adjust the text accordingly. Additionally, forthcoming research endeavors could incorporate named entity recognition to identify a comprehensive list of disease or symptom-related terms as vocabulary prior to applying the TF-IDF approach. This strategy aims to encompass multi-word phrases that accurately convey the true essence of clinical terms. Lastly, it is worth considering the utilization of bidirectional encoder representations from transformers or large language models [40] in future studies. These models have the capability to capture the semantic meaning embedded within clinical notes, potentially leading to more precise predictions.

## Conclusions

With the increasing number of patients seeking emergency care, ED overcrowding has become a global issue that requires alleviation. The main objective of this study is to utilize the ensemble learning method to establish an ED disposition prediction model that will allow ED clinicians to predict patient disposition outcomes early-on. The study integrates structured and unstructured data to enhance the predictive capability of the given model. The developed predictive model can provide ED clinicians with the ability to predict patient discharge outcomes as soon as possible, with the aim of mitigating ED over-crowding. Additionally, this study employs LIME to explain how the predictive model forecasts ED disposition and enables ED clinicians to reference and implement appropriate interventions to enhance patient care.

### Electronic supplementary material

Below is the link to the electronic supplementary material.

Supplementary Material 1

## Data Availability

The datasets used and analyzed during the current study are available from the corresponding author on reasonable request. The source codes supporting the findings of this study can be accessed at: https://osf.io/fdrta/.

## Abbreviations

ACC: Accuracy
AUC/AUROC: Area under the receiver operating characteristic curve
AUPRC: Area under the precision-recall curve
BOW: Bag-of-words
CNN: Convolutional neural network
COVID-19: Coronavirus disease 2019
CT: Computed tomography
DNN: Deep neural network
DT: Decision tree
ED: Emergency department
FN: False negative
FP: False positive
GB: Gradient boosting
GBM: Gradient boosting machine
ICD-10-CM: International classification of diseases, 10th edition, clinical modification
ICU: Intensive care unit
IQR: Interquartile range
KNN: K-nearest neighbors
LIME: Local interpretable model agnostic explanations
LR: Logistic regression
LSTM: Long-short term memory
MAE: Mean absolute error
ML: Machine learning
MLP: Multilayered perceptron
MNB: Multinomial Naïve Bayes
MNN: Multilayered neural network
NPV: Negative predictive value
PPV: Positive predictive value
RF: Random forest
PREC: Precision
RECA: Recall
RNN: Recurrent neural network
RT: Randomized tree
SD: Standard deviation
SENS: Sensitivity
SOAP: Subjective, objective, assessment, and plan
SPEC: Specificity
SVM: Support vector machine
TF-IDF: Term-frequency-inverse document frequency
TN: True negative
TP: True positive
TTAS: Taiwan triage and acuity scale
XGBoost/XGB: eXtreme gradient boosting
