# Supplementary material for: An ensemble model for predicting dispositions of emergency department patients
Source: BMC Med Inform Decis Mak. 2024 Apr 22;24:105. doi: 10.1186/s12911-024-02503-5 (PMC11036695; doi:10.1186/s12911-024-02503-5)
Supplement: Supplementary file 1 — Supplementary Material 1 [file 12911_2024_2503_MOESM1_ESM.docx]

# Appendices

Supplementary file A. Prior studies using unstructured data for predicting emergency disposition

| Study | Disposition | Features | | ML algorithm | Performance | | |
| --- | --- | --- | --- | --- | --- | --- | --- |
|  |  | Structured | Unstructured |  | Structured | Unstructured | Combined |
| Lucini et al. [12] | Hospitalization |  | SOAP framework notes | DT, RF, Extremely RT, AdaBoost, LR, MNB, SVM, Nu-SVM |  | F1: 0.777, RECA: 0.8248, PREC: 0.7347 |  |
| Zhang et al. [20] | Hospital admission / transfer | Demographics, vital signs, triage variables, comorbidities, injury | Reasons for visit or cause of injury | LR, MNN | AUC: 0.824 |  | AUC: 0.846 |
| Sterling et al. [11] | Admission, transfer, in-ED death |  | Nursing triage notes | DNN |  |  | AUC: 0.785, F1: 0.572, ACC: 0.725, SENS: 0.693, SPEC: 0.736, PPV: 0.486, NPV: 0.870 |
| Arnaud et al. [16] | Hospitalization | Demographics, vital signs | Triage-provided observations (notes), psychiatric history, surgical history, medical history | MLP, CNN, ensemble |  |  | AUC: 0.83 |
| Chen et al. [21] | Hospitalization | Demographics, vital signs | Physicians' clinical notes | LR, DNN |  |  | AUC: 0.862,  F1: 0.602, SENS: 0.6597,  SPEC: 0.0.8749 |
| Fernandes et al. [22] | Mortality or cardiopulmonary arrest | Demographics, vital signs | Chief complaint | LR, RF, XGBoost |  |  | AUROC: 0.96,  AUPRC: 0.31, Average PREC: 0.30, RECA: 0.84, SPEC: 0.94, PREC: 0.06, F1: 0.12 |
| Joseph et al. [23] | Mortality or ICU admission | Demographics, vital signs | Chief complaint | LR, RNN, XGBoost |  |  | AUC: 0.857,  SENS: 0.845,  SPEC: 0.704 |
| Tahayori et al. [10] | Admit to ward |  | Triage notes | DNN |  | ACC: 0.83, AUC: 0.88, SENS: 0.72, SPEC: 0.86, PREC: 0.56, F1-score: 0.63 |  |
| Roquette et al. [24] | Admission to hospital | Demographics, vital signs | Chief complaint, drugs, triage notes, past visit, triage notes, past visit chief complaint | Catboost |  |  | AUC: 0.891 |
| Butler et al. [25] | COVID-19 infection | Demographics, vital signs, comorbidities | Chest radiographs | LightGBM, CNN | AUC: 0.79, SENS: 0.7, SPEC: 0.76 | AUC: 0.71, SENS: 0.56, SPEC: 0.69 |  |
|  | Acute respiratory distress syndrome | Demographics, vital signs, comorbidities | Chest radiographs | LightGBM, CNN | AUC: 0.753, SENS: 0.6, SPEC: 0.75 | AUC: 0.74, SENS: 0.63, SPEC: 0.73 | AUC: 0.78, SENS: 0.53, SPEC: 0.71 |
|  | ICU admission | Demographics, vital signs, comorbidities | Chest radiographs | LightGBM, CNN | AUC: 0.675, SENS: 0.65, SPEC: 0.65 | AUC: 0.67, SENS: 0.63, SPEC: 0.58 | AUC: 0.68, SENS: 0.58, SPEC: 0.72 |
|  | Risk of mortality at time of ED admission | Demographics, vital signs, comorbidities | Chest radiographs | LightGBM, CNN | AUC: 0.755, SENS: 0.72, SPEC: 0.68 | AUC: 0.76, SENS: 0.62, SPEC: 0.71 | AUC: 0.758, SENS: 0.67, SPEC: 0.71 |
| Dayan et al. [26] | Death | Demographics, vital signs, laboratory data | X-rays | DNN |  |  | AUC: 0.956  SENS: 0.950  SPEC: 0.882 |
| Klang et al. [17] | Admission to ICU | Demographics, chief complaints, comorbidities, vital signs, laboratory data | Nursing and physician test notes | Ensemble | AUC: 0.92 | AUC: 0.90 | AUC: 0.93 |
| Klang et al. [18] | In-hospital mortality | Demographics, vital signs, comorbidities, laboratory orders, previous ED visits and hospitalizations, number of days to previous visits, type of ward if hospitalized | Nursing and physician notes, flowsheet records, chief complaints | LR, GB, Ensemble |  |  | AUC: 0.98, SENS: 0.39, SPEC: 0.99, PPV: 0.20, NPV: 1, F1: 0.26 |
| Di Napoli et al. [27] | Mortality | Clinical data, laboratory data | Chest CT | CNN |  |  | AUC: 0.96, ACC: 0.927, SENS: 0.905, SPEC: 0.937 |
|  | Intubation | Clinical data, laboratory data | Chest CT | CNN |  |  | AUC: 0.95, ACC: 0.913, SENS: 0.915, SPEC: 0.898 |
|  | ICU admission | Clinical data, laboratory data | Chest CT | CNN |  |  | AUC: 0.94, ACC: 0.896, SENS: 0.90, SPEC: 0.865 |
| Duanmu et al. [28] | Mortality | Demographics, vital signs, comorbidities, laboratory data | Chest X-ray | CNN | AUC: 0.80,  ACC: 0.80,  PREC: 0.77,  RECA: 0.70, F1 score: 0.71 | AUC: 0.83,  ACC: 0.82,  PREC: 0.82,  RECA: 0.69, F1 score: 0.74 | AUC: 0.87,  ACC: 0.85,  PREC: 0.80,  RECA: 0.68, F1 score:0.74 |
|  | Duration on invasive mechanical ventilation | Demographics, vital signs, comorbidities, laboratory data | Chest X-ray | CNN | MAE: 2.88 | MAE: 3.11 | MAE: 2.56 |
| Patel et al. [15] | Hospitalization | Demographics, laboratory data, medical history, vitals | Triage notes | XGBoost |  |  | AUC: 0.875, SENS: 0.454, SPEC: 0.958,  PREC: 0.712 |
| Bunney et al. [14] | Likelihood of hospitalization | Demographics, triage, vital signs, geriatric intervention | Chief complaints | LR, RF, GB, NN, KNN, SVM |  |  | AUC: 0.826, SENS: 0.787 SPEC: 0.701 PPV: 0.792 NPV: 0.696 |
| Chen et al. [13] | In-hospital cardiac arrest or ICU admission | Demographic data, vital signs | Chief complaints, present illness, past medical history | NB, RF, XGB, LR, MLP, CNN, LSTM, DNN |  |  | AUROC: 0.874, AUPRC: 0.207, SENS: 0.499, SPEC: 0.933, PPV: 0.161, NPV: 0.986 |

*Notes:*

1. ACC: Accuracy, AUC/AUROC: Area under the receiver operating characteristic curve, AUPRC: Area under the precision-recall curve, MAE: Mean absolute error, ML: Machine learning, NPV: Negative predictive value, PPV: Positive predictive value, PREC: Precision, RECA: Recall, RT: Randomized tree, SENS: Sensitivity, SPEC: Specificity.
2. CNN: Convolutional neural network, DNN: Deep neural network, DT: Decision tree, GB: Gradient boosting, GBM: Gradient boosting machine, KNN, K-nearest neighbors, LR: Logistic regression, LSTM: Long short-term memory, MLP: Multilayer perceptron, MNB: Multinomial Naïve Bayes, MNN: Multilayer neural network, RF: Random forest, RNN: Recurrent neural network, SVM: Support vector machine, XGBoost/XGB: eXtreme gradient boosting.
3. CT: Computed tomography, ED: Emergency department, ICU: Intensive care unit, SOAP: Subjective, objective, assessment, and plan.

Supplementary file B. Measures used in this study

| Metric | Formula/Algorithm |
| --- | --- |
| Accuracy | $\frac{TP+TN}{TP+FN+FP+TN}$ |
| AUROC | See *Note* 3 |
| Precision | $\frac{TP}{TP+FP}$ |
| Recall | $\frac{TP}{TP+FN}$ |
| F1 score | $\frac{2*Precision*Recall}{Precision+Recall}$ |

*Note:*

1. AUROC denotes area under the receiver operating characteristic curve.
2. *TP* means true positive, *TN* means true negative, *FP* means false positive, *FN* means false negative.
3. The AUROC was calculated using Algorithm 1 (p. 866) and Algorithm 2 (p. 869) as described in the study by Fawcett (2006).

Fawcett, T. (2006). An introduction to ROC analysis. *Pattern Recognition Letters*, *27*(8), 861-874. https://doi.org/10.1016/j.patrec.2005.10.010
